# Proline Affects Flowering Time in Arabidopsis by Modulating FLC Expression: A Clue of Epigenetic Regulation?

**DOI:** 10.3390/plants11182348

**Published:** 2022-09-08

**Authors:** Roberto Mattioli, Antonio Francioso, Maurizio Trovato

**Affiliations:** 1 Department of Biochemical Sciences, Sapienza University of Rome, 00185 Rome, Italy; 2 Instituto Universitario de Bio-Orgánica Antonio González, 38200 San Cristóbal de La Laguna, Spain; 3 Department of Biology and Biotechnology, Sapienza University of Rome, 00185 Rome, Italy

**Keywords:** *FLC*, flowering time, proline, *p5cs1 p5cs2/P5CS2*, reactive nitrogen species, reactive oxygen species

## Abstract

The recent finding that proline-induced root elongation is mediated by reactive oxygen species (ROS) prompted us to re-evaluate other developmental processes modulated by proline, such as flowering time. By controlling the cellular redox status and the ROS distribution, proline could potentially affect the expression of transcriptional factors subjected to epigenetic regulation, such as *FLOWERING LOCUS C* (*FLC*). Accordingly, we investigated the effect of proline on flowering time in more detail by analyzing the relative expression of the main flowering time genes in *p5cs1 p5cs2/P5CS2* proline-deficient mutants and found a significant upregulation of *FLC* expression. Moreover, proline-deficient mutants exhibited an adult vegetative phase shorter than wild-type samples, with a trichome distribution reminiscent of plants with high *FLC* expression. In addition, the vernalization-induced downregulation of *FLC* abolished the flowering delay of *p5cs1 p5cs2/P5CS2*, and mutants homozygous for *p5cs1* and *flc-7* and heterozygous for *P5CS2* flowered as early as the *flc-7* parental mutant, indicating that FLC acts downstream of *P5CS1*/*P5CS2* and is necessary for proline-modulated flowering. The overall data indicate that the effects of proline on flowering time are mediated by FLC.

## 1. Introduction

The finding that proline modulates root elongation by controlling the abundance and distribution of O_2_^•−^ and H_2_O_2_ in the root meristem [1] raises the possibility that other developmental processes affected by proline [2,3,4,5,6,7] may rely on redox balance and ROS signaling. Indeed, an increasing body of evidence indicates that the redox state of the cell is a critical determinant for cell proliferation, as well as for the activity of shoot and root meristems and cell cycle progression [8]. ROS signaling, triggered in turn by redox unbalance, is also known to affect several developmental processes, including wall loosening and cell expansion [9], fruit development [10], root meristem size [1,11,12,13,14], xylem differentiation [15], germination and dormancy [16,17], pollen development [18,19], and time of flowering [2,4,20].

Contrary to root elongation, embryo development, and pollen development, where the involvement of proline is more consolidated and better understood, flowering time is more vaguely linked to proline, and a convincing model to explain the effect of this amino acid on flowering time is still lacking.

The current view of floral signaling has identified six major signaling pathways, namely the photoperiodic, vernalization, ambient temperature, age, autonomous, and gibberellin-dependent pathways [21,22,23,24,25,26], which, in response to exogenous cues (photoperiodic, ambient temperature, and vernalization pathways) and autogenous cues (age, autonomous and gibberellin-dependent pathways), induce the transformation of a shoot apical meristem (SAM) from a vegetative to inflorescence meristem.

Extensive physiological, genetic, and genome-wide research, mainly based on Arabidopsis flowering time mutants, has established that floral pathways converge to a few floral integrator genes to ultimately activate the floral meristem identity genes. Different floral pathways, triggered by environmental, developmental, and hormonal stimuli, are integrated into a complex network, including transcription factors, microRNAs, mobile factors, hormones, and chromatin-modifying proteins, to finely tune floral transition and maximize reproductive fitness [26,27].

Most Arabidopsis ecotypes, for example, are facultative long-day responders and, as soon as the days become long enough, initiate the transition from a vegetative to an inflorescence meristem. Irrespective of the day length, however, no flowering is possible during the juvenile vegetative phase (an early vegetative stage characterized by rosette leaves with adaxial trichomes), and only after transition to the adult vegetative phase (a late vegetative stage characterized by rosette leaves with both adaxial and abaxial trichomes) does the plant become competent to flower [28].

Similarly, in most Arabidopsis ecotypes, flowering is possible only after a long period of cold temperature, no matter how long the day is [29]. From a molecular point of view, all floral pathways work similarly by activating signaling cascades that lead to the downregulation of genes that repress flowering, such as *FLOWERING TIME C* (*FLC*), and the upregulation of genes that promote flowering, such as the floral integrator genes *FLOWERING TIME T* (*FT*), *LEAFY* (*LFY*), or *SUPPRESSOR OF CONSTANS 1* (*SOC1*). All floral pathways eventually converge to activate the floral meristem identity genes, such as *APETALA1* (*AP1*), *LFY*, or *CAULIFLOWER* (*CAL*), fully accomplishing the transition from a vegetative to a floral SAM.

Despite the rapid and abundant accumulation of free proline observed by many authors during and after flower transition [30,31], relatively few reports have described the effects on flowering time due to the genetic manipulation of proline metabolic genes [2,4,20,32,33]. The proline released from other sources seems also capable to affect flowering time, as shown by Zdunek-Zastocka et al., (2027), who overexpressed a transgenic proline aminopeptidase, an enzyme that releases proline from the N-termini of peptides, in Arabidopsis, leading to proline accumulation and early flowering [34]. Overall, however, little information is available, as yet, on the molecular and genetic details of this process, and it is not clear which genes are involved in proline-modulated flowering or with which floral pathway(s) proline interacts.

The possible involvement of *FLC*, though, has been suggested by Guan et al., (2019), who overexpressed the gene *Δ1-pyrroline-5-carboxylate synthetase* (*P5CS*), the rate-limiting enzyme of proline biosynthesis in higher plants, in switchgrass (*Panicum virgatum* L.) and analyzed the expression of the transgenic plants using RNA-seq. They found, among the lines with high levels of proline, others with low proline levels characterized by stunted growth, severe late flowering, and upregulation of *FLC*. Although the RNA-seq analysis of the transgenic lines revealed other genes with expressions significantly different from those of the wild-type samples, the data on *FLC* are interesting because they correlate well with the flowering time of the mutant lines. Since *FLC* is subjected to epigenetic regulation [35], which is known to be controlled by the redox status of the cell [36], the effects of proline on flowering time can potentially rely on a redox-dependent epigenetic regulation of *FLC*. Here, we investigate the flowering time genes with which proline interacts, using a combination of genetic and molecular techniques, and report that, in Arabidopsis, the effects of proline on flowering time requires *FLC*, suggesting a possible redox-mediated epigenetic control of proline on flowering time.

## 2. Results

### 2.1. FLC Is Upregulated in the Proline Biosynthesis Mutant p5cs1 p5cs2/P5CS2

To investigate the interactions between flowering time and proline in Arabidopsis, we analyzed the expression of representative flowering time genes in the late-flowering *p5cs1 p5cs2/P5CS2* quasi-double mutant [4]. As detailed in Table 1, we chose genes belonging to the main floral pathways, including the photoperiodic pathway, the autonomous pathway, the vernalization pathway, and the GA-dependent pathway. We also tested the expression of the integrator genes *LFY*, *FT*, *FLC*, and *SOC1*, and the floral meristem identity genes *AP1* and *CAL*.

We analyzed the expression of these genes via semi-quantitative RT-PCR, either in shoot apices or in leaves, depending on the site of expression of each gene inferred by the scientific literature. In particular, we analyzed the expression of *CO*, *GI*, *FWA*, *GA1*, *GA3ox1*, *GA3ox2*, *GA3ox3*, *GA3ox4*, *GA20ox1*, *GA20ox2*, and *GA2ox1* in the leaves, and the expression of *LD*, *FCA*, *FLK*, *FLD*, *FPA*, *FVE*, *FY*, *VRN1*, *VRN2*, *VIN3*, *LFY*, *FT*, *SOC1*, *AP1*, and *CAL* in the shoot apices. The expression of *FLC*, however, was investigated both in shoot apices and leaves. We germinated and potted all the plantlets simultaneously and took all the samples when the first floral bud was visible in the control wild-type population.

**Figure 1:**
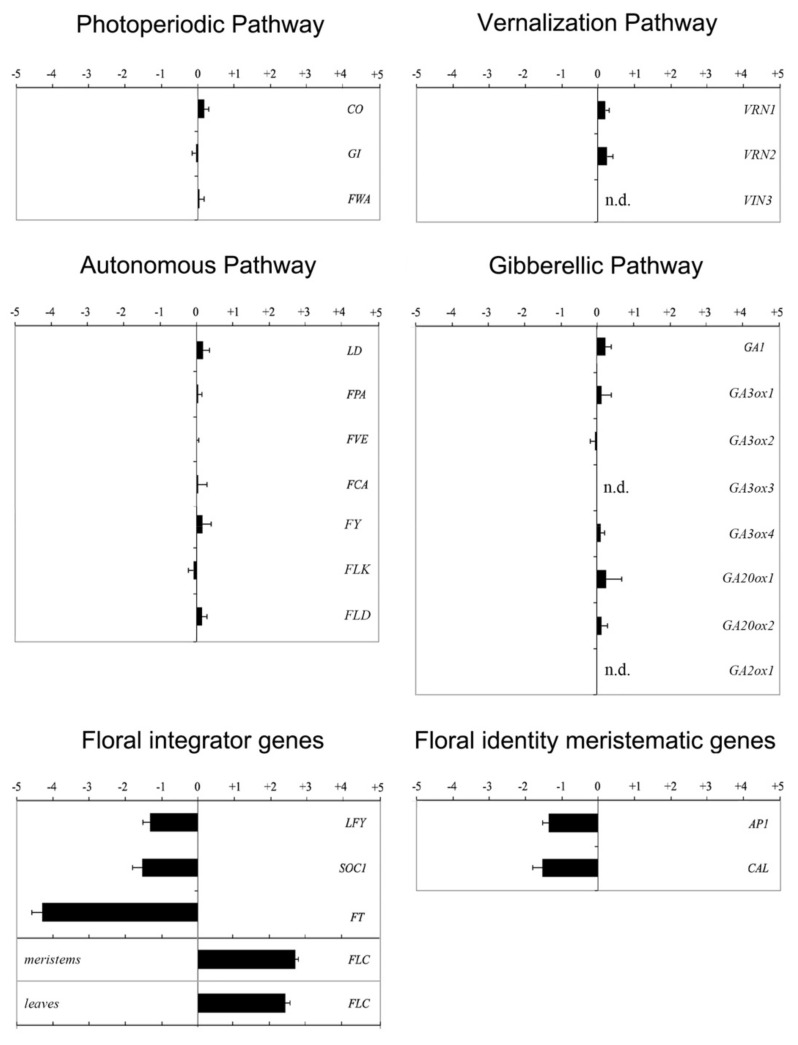
Relative expression of representative flowering time genes in shoot apices and leaves of *p5cs1 p5cs2/P5CS2* performed via semi-quantitative RT-qPCR. RNA for cDNA synthesis was extracted from either apical shoots (*SOC1*, *FT*, *FLC*, *LFY*, *AP1*, and *CAL*) or leaves (*CO* and *FLC*). The constitutive housekeeping gene *ACT8* was used as reference control. Error bars indicate standard errors. All data are means from three independent experiments.

We found a significant upregulation of *FLC*, expressed four times as high in the proline-deficient mutant than in wild-type samples, and downregulation of genes downstream of *FLC*, such as *SOC1*, *FT*, *LFY*, *AP1*, and *CAL* (Figure 1). On the contrary, we saw no significant variations in the expression level of genes upstream to *FLC*.

**Figure 2:**
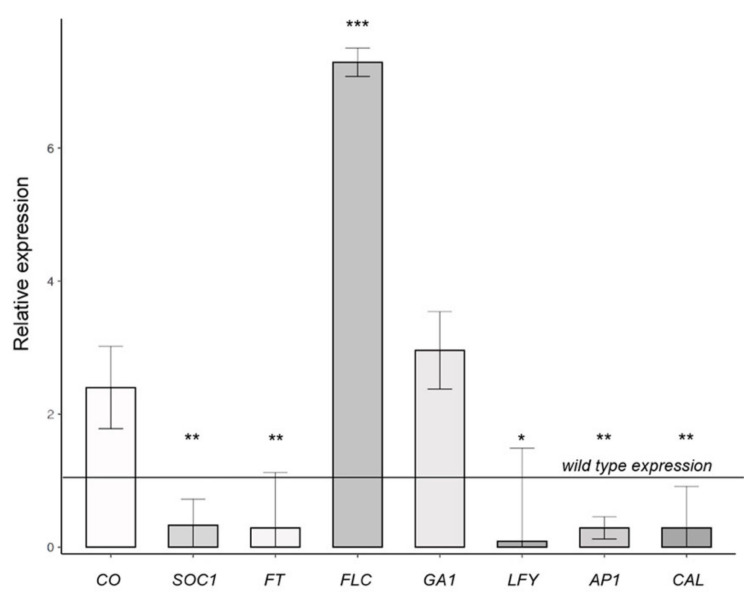
Relative expression of representative flowering time genes in shoot apices and leaves of *p5cs1 p5cs2/P5CS2*. RT-qPCR was performed on cDNA from either apical shoots (*SOC1*, *FT*, *FLC*, *LFY*, *AP1*, and *CAL*) or leaves (*CO* and *FLC*). The constitutive housekeeping gene *ACT8* was used as reference control to normalize the RT-qPCR. Error bars indicate standard deviation (SD). All data are means from three independent experiments. For sake of simplicity, the plot reports only the fold change of the genes expressed in the mutant relative to wild type indicated by the straight line. Consequently, the SD of the wild-type expression is not shown on the plot but contributes to determining statistical significance in pairwise statistical testing (* *p* < 0.05; ** *p* > 0.01; *** *p* < 0.001). Calculations of ΔΔCq and Welch’s t-test were performed with the free “R” software [37] using the R package “PCR” [38].

To confirm and extend these data, using real-time RT-qPCR (Figure 1), we further analyzed the most relevant results of the semi-quantitative analysis (*FLC*, *CO*, *SOC1*, *FT*, *GA1*, *LFY*, *AP1*, and *CAL*). As shown in Figure 2, the real-time RT-qPCR essentially confirmed a strong upregulation of the floral repressor *FLC* and downregulation of *SOC1*, *FT*, *LFY*, *AP1*, and *CAL*. *FLC*, in particular, appeared to be the gene most strongly affected by proline deficiency, as in *p5cs1 p5cs2/P5CS2*, its average expression was statistically significant (*p* < 0.001) and about seven times as high as that in wild-type samples.

### 2.2. Proline-Deficient Mutants Exhibit a Trichome Distribution Reminiscent of Plants Overexpressing FLC

In addition to being late-flowering, plants with high *FLC* levels have a prolonged adult vegetative phase. Willmann and Poethig (2011) [39] compared the timing and distribution of trichomes, a reliable marker of phase transition, between Columbia and *FRI FLC*, a Columbia line containing both *FRI*- and *FLC*-active alleles [40]. In *FRI FLC* plants, they found many adult vegetative leaves containing one or more trichomes on their abaxial face, while no differences were found in the timing of abaxial trichome production (i.e., juvenile vegetative to adult vegetative transition phase). These results indicated that the late-flowering phenotype of the *FRI FLC* plants has to be accounted for by a prolonged adult vegetative phase [39]. Since in *p5cs1 p5cs2/P5CS2*, the expression of *FLC* is higher than in wild-type samples, we wondered whether the late-flowering phenotype of this mutant might rely on a prolonged adult vegetative phase, as in the case of *FRI FLC*. To assess this point, we analyzed the trichome disposition of leaves of non-vernalized *p5cs1 p5cs2/P5CS2*, grown under long day conditions (LD) and found that the trichome distribution of *p5cs1 p5cs2/P5CS2* rosette leaves resembles that of plants overexpressing *FLC*. Although the delay of flowering exhibited in LD by *FRI FLC* [40] was much higher compared with *p5cs1 p5cs2/P5CS2*, in both mutants, the late-flowering phenotype was not affected by the duration of the juvenile vegetative phase but was accounted for by an adult vegetative phase longer than wild-type samples.

**Figure 3:**
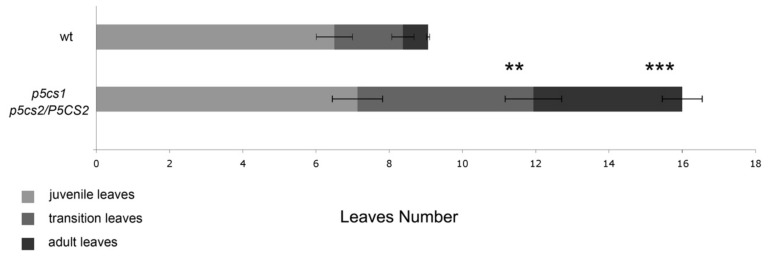
Graphic representation of the leaf types present in wt and proline mutants classified based on the presence of trichomes on either the adaxial or abaxial side. The upper horizontal bar represents the trichome distribution in wild type. The lower bar represents the trichome distribution on *p5cs1 p5cs2/P5CS2*. Juvenile leaves are defined as rosette leaves with adaxial trichomes only. Transition leaves are defined as rosette leaves with at least one trichome located at the abaxial base of the midrib. Adult leaves are defined as rosette leaves with abaxial trichomes distributed all over the abaxial side, both at the midrib and at the lamina of the leaf. All the experiments were performed as triplicate independent experiments, and results are expressed as average values ± SE. Statistical significance was assessed with a Welch two-sample *t*-test (** *p* < 0.01; *** *p* < 0.001).

As shown in Figure 3, the number of juvenile leaves containing only adaxial trichomes (Figure 3, the light gray portion of the bars) was not significantly different between wild-type and *p5cs1 p5cs2/P5CS2* plants, with an average number of juvenile leaves of 6.2 ± 0.41 and 7.1 ± 0.76 for wild-type and *p5cs1 p5cs2/P5CS2* samples, respectively. On the contrary, the number of adult vegetative leaves, including both transition and adult leaves, bearing trichomes on the leaf abaxial side (Figure 2, the black portion of the bars), was significantly higher in *p5cs1 p5cs2/P5CS2* (8.85 ± 1.43) than in wild-type samples (2.7 ± 0.54), with a high degree of statistical confidence (*p* < 0.01 for transition leaves and *p* < 0.001 for adult leaves). In particular, we counted 4.85 ± 0.82 transition leaves (Figure 2, the gray portion of the right bar) and 4 ± 0.61 adult leaves (Figure 3, the dark gray part of the right bar) in *p5cs1 p5cs2/P5CS2* and 1.85 ± 0.34 (Figure 3, the gray portion of the left bar) transition leaves and 0.85 ± 0.14 adult leaves (Figure 3, the dark gray part of the left bar) in Col-0 wild type. Overall, these data suggest that the delay of flowering of proline-deficient mutants may be correlated to elevated levels of *FLC* expression.

### 2.3. Vernalization Experiments Show That FLC Is Necessary for Proline-Mediated Flowering

FLC is a powerful floral repressor that functions by repressing the expression of floral inducers *FT* and *SOC1* by directly binding to their promoters [41,42,43]. As long as *FLC* levels remain high, flowering is repressed. It is known, however, that a period of prolonged cold (vernalization) activates the genes of the vernalization pathway to switch off the *FLC* expression and, in turn, promote flowering [23].

If the upregulation of *FLC* is related to the prolonged adult vegetative phase and, in turn, to the late-flowering phenotype of the *pc5s1 p5cs2/P5CS2* plants, vernalization treatment should, in principle, rescue the late-flowering phenotype of the proline-deficient mutants.

**Figure 4:**
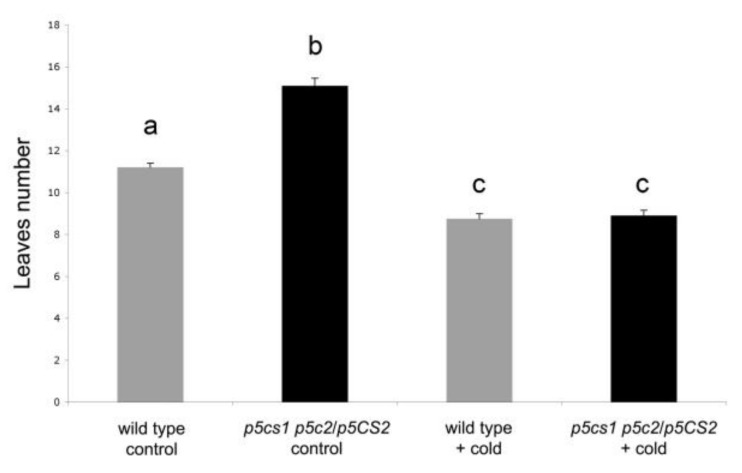
Vernalization abolishes the late-flowering phenotype of the *p5cs1 p5cs2/P5CS2* mutant. After vernalization treatment at 4 °C for three months, the late-flowering phenotype of the low-proline mutant was complemented, and the mutant became as early flowering as a vernalizated wild type. Vertical bars represent the mean number of rosette leaves +/− SE at the time of flower bud emergence. A two-way ANOVA analysis confirmed a significant effect of vernalization on flowering time. Pairwise comparisons with Tukey post hoc correction were used to analyze differences between individual samples. Different letters indicate statistically different group means. All pairwise comparisons were significant at *p* < 0.001, except the difference between wild-type-vernalized and *p5cs1 p5cs2/P5CS2*-vernalized samples, which was non-significant. Each box represents the mean of two independent experiments, each one comprising 10 plants.

To evaluate this hypothesis, seeds from *pc5s1 p5cs2/P5CS2* mutants and wild-type controls were vernalized for three months at 4 °C in the dark. As shown in Figure 4, both the wild-type and *pc5s1 p5cs2/P5CS2* plants responded to the vernalization treatment by flowering earlier than in non-vernalized conditions. The late-flowering phenotype of the *pc5s1 p5cs2/P5CS2* plants, in particular, appeared to be completely rescued as, after vernalization, the mutant plants flowered as early as wild-type plants, at 8.94 ± 0.25 and 8.75 ± 0.24 rosette leaves, respectively. This result suggests that proline does not participate in the vernalization pathway and indicates that *FLC* is necessary for proline-mediated flowering.

### 2.4. Flc-7 p5cs1 p5cs2/P5CS2 Mutants Flower as Early as the Flc-7 Parental Mutant

To further confirm the role of *FLC* in proline-modulated flowering and to assess the epistatic relationships between proline and *FLC*, we generated an *flc p5cs1 p5cs2/P5CS2* mutant by crossing *flc* with *p5cs1 p5cs2/P5CS2*.

As all the information on the effects of proline on flowering has been gained in Col-0, and since *flc-3*, the most characterized *FLC* mutant in Columbia, bears a point mutation in its coding sequence and is difficult to follow in genetic crosses, we characterized a novel *FLC* mutant in Columbia to generate the *flc-7 p5cs1 p5cs2/P5CS2* mutant. A T-DNA insertion mutant (GK 145D01) from the GABI-KAT collection [44] was found and obtained from the NASC stock center. Heterozygous individuals, carrying a single T-DNA insertion, were selected through the segregation analysis of the T-DNA-born sulfadiazine resistance and were allowed to self-pollinate to isolate homozygous mutants. Homozygous lines, identified by PCR analysis and verified by the sequencing of the PCR products, confirmed the presence of a T-DNA insertion in the first intron of the *FLC* gene, located 2881 bp downstream of the ATG codon (Figure 4a). No transcription of the *flc* mutant allele, hereafter referred to as *flc-7*, was found in RT-PCR experiments (Figure 4b), indicating that *flc-7* is a knockout allele of *FLC.* To characterize the *flc-7* phenotype, we focused our attention on the most distinguishing features of known *flc* alleles, i.e., the anticipation of flowering and the short adult vegetative phase, relative to wild type. As visible in Figure 4c,d, the *flc-7* mutants behaved as early flowering with an average number (8.29 ± 0.13) of rosette leaves at the time of flowering, significantly (*p* < 0.001) less than the number (11.45 ± 0.28) of rosette leaves of the Col-0 wild-type control. Most importantly, while both *flc-7* and Col-0 showed, on average, the same number of juvenile vegetative leaves, only one leaf (0.58 ± 0.14) with adult vegetative features was present, on average, on *flc-7* mutants at flowering, compared with 2.3 ± 0.21 leaves of control plants. Overall, *flc-7* was found to be a novel knockout allele of *FLC* in Col-0, with a phenotype similar to *flc-3.*

To verify the role of *FLC* in proline-modulated flowering and to assess the epistatic relationships between proline and *FLC*, we crossed the newly characterized *flc-7* with *p5cs1 p5cs2/P5CS2*. As proline-deficient mutants are male sterile [5,6], *flc-7* was used as a pollen donor to fertilize emasculated *p5cs1 p5cs2/P5CS2*.

**Figure 5:**
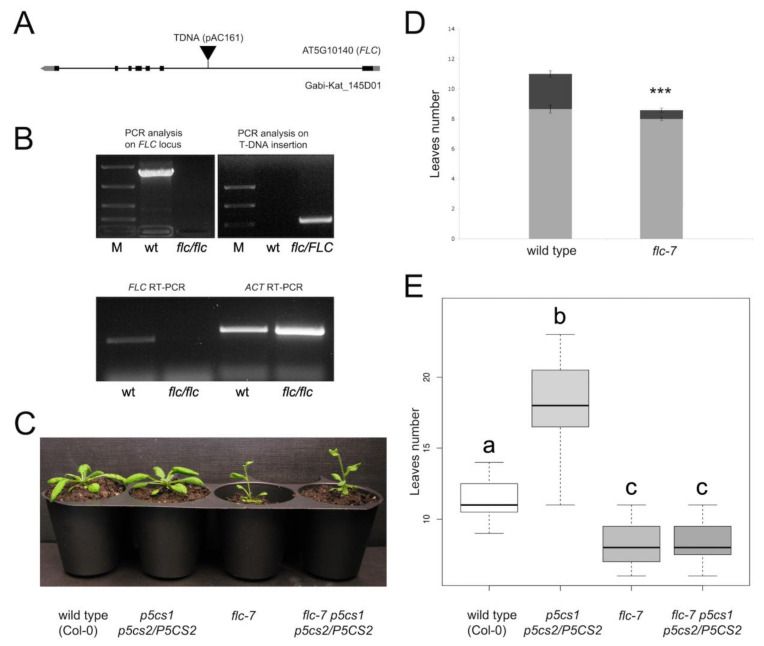
Characterization of *flc-7*, a knockout allele of *FLC*, and its epistatic relation with *p5c1p5cs2/P5CS2*: (**A**) localization of the Gabi-Kat_145D01 T-DNA insertion in the *FLC* gene; (**B**) molecular characterization of the homozygous *flc-7* mutant; (**C**) phenotype of wild type (Col-0), *p5cs1 p5cs2/P5CS2*, *flc-7*, and *flc-7 p5cs1 p5cs2/P5CS2* at flowering time showing that the quasi-triple mutant is as early flowering as *flc-7;* (**D**) bar plots showing the number of rosette leaves at flowering time in wt (left), and flc-7 (right). The lower light gray part of the bar shows juvenile leaves, while the upper dark gray portion of the bar represents adult leaves. Statistical significance was assessed with a Welch two-sample t-test (*** *p* < 0.001) (**E**) box plot representing the mean number of rosette leaves at floral transition in wild type (Col-0), *p5cs1 p5cs2/P5CS2*, *flc-7*, and *flc-7 p5cs1 p5cs2/P5CS2*. A one-way ANOVA, followed by a Tukey post hoc test found no significant differences between *flc-7* and *flc-7 p5cs1 p5cs2/P5CS2*. Different letters indicate statistically different means (*p* < 0.001 between a-b, a-c, b-c). Each box represents the mean of three independent experiments, each one including 10 plants.

As shown in Figure 5, the resulting *flc-7 p5cs1 p5cs2/P5CS2* mutant, homozygous for *flc-7* and *p5cs1* and heterozygous for *p5cs2*, behaved as early flowering with an average number of leaves of 8.71 ± 0.20 at the time of flowering, with as few rosette leaves as the *flc-7* parental line (8.45 ± 0.19). In striking contrast, the other parental line, *p5cs1 p5cs2/P5CS2*, flowered late, with an average of 17.50 ± 2.22 rosette leaves. A one-way ANOVA followed by a Tukey post hoc test confirmed that the average number of leaves of *flc-7 p5cs1 p5cs2/P5CS2* was significantly different from both those of wild-type and *p5cs1 p5cs2/P5CS2* (*p* < 0.001) samples but not significantly different from those of *flc-7* (*p* < 0.995). These data clearly show that *FLC* acts downstream of *P5CS1*/*P5CS2* and is necessary for proline-modulated flowering.

## 3. Discussion

In light of the recently discovered interactions between proline and ROS in the modulation of root meristem size, we re-analyzed the genetic and molecular mechanisms of the effects of proline on flowering time in search of its molecular basis. By controlling the cellular redox status and the ROS distribution in the apical meristem, proline can potentially affect the expression of transcriptional factors subjected to epigenetic post-transcriptional or post-translational regulations.

A thorough semi-quantitative analysis of representative flowering time genes, followed by a quantitative RT-qPCR analysis of the most significant genes, revealed a significant upregulation of the floral repressor *FLC* in the leaves and shoot apices of *p5cs1 p5cs2/P5CS2* compared with the wild-type control. Not surprisingly, we also found a downregulation of floral integrator genes, and floral meristem identity genes downstream of *FLC*.

To support this finding, we analyzed the trichome distribution of *p5cs1 p5cs2/P5CS2* and wild types since it is known that plants overexpressing *FLC* have longer transition and adult phases characterized by abaxial trichomes [39]. As shown in Figure 3, the developmental phase progression of *p5cs1 p5cs2/P5CS2* resembled that of plants overexpressing *FLC*, such as *FRI:FLC* or *CaMV35S::FLC* grown over long days [39]. Furthermore, we showed that a prolonged cold treatment turned *p5cs1 p5cs2/P5CS2* from late flowering to early flowering. Similarly, vernalization promotes flowering in the late-flowering ecotypes by decreasing the level of *FLC* transcript [35]. Moreover, a quasi-triple mutant between *flc-7*—the newly characterized *flc allele*—and *p5cs1 p5cs2/P5CS2* is as early flowering as *flc-7*. Overall, these experiments indicate that the late-flowering phenotype of *p5cs1 p5cs2/P5CS2* relies on *FLC* upregulation and that FLC is required for the late flowering of proline mutants.

This finding is in agreement with the work of Guan et al. (2019) [20], who generated switchgrass plant transgenics for *P5CS* and found that the lines with low proline levels correlate with late flowering and *FLC* upregulation. Intriguingly, profilins, a class of small molecules involved in actin binding and organization capable of binding poly-proline stretches and proline-rich proteins, have been shown to affect flowering time [45]. Moreover, ectopic expression in the tobacco of *GhPRF1*, a cotton profilin gene, resulted in early flowering, downregulation of *FLC*, and upregulation of *AP1*, *SOC1*, and *FT1* [46].

The involvement of FLC in proline-mediated flowering may suggest a link between redox regulation and the epigenetic regulation of flowering. Indeed, recent studies suggest that the redox status of the cell affects the enzymes involved in plant epigenetic modifications, such as DNA methylation and histone protein acetylation, leading, in turn, to chromatin remodeling and differential gene expression [35]. In a comparative expression study between the wild-type and redox-compromised mutant *stn7* (*state transition 7*), for example, Dietzel et al., (2015) [47] found no or little expression of many nuclear genes in *stn7* mutants exposed to high-light treatments. Furthermore, in wild-type plants, redox signals generated from the chloroplast were found to enhance the activity of histone acetyl-transferase (HAT) and histone deacetylase (HDAC), two key enzymes of epigenetic regulation. Moreover, Rai et al., (2018) [48] reported that in *Lablab purpureus*, the increase in O_2_^•−^ and H_2_O_2_ caused by high-temperature stress can be relieved by the combined application of salicylic acid (SA) and sodium nitroprusside (SNP)—a nitric oxide (NO) donor. Notably, the supplementation of SNP and NO to temperature-stressed plants led to increased activity of antioxidant enzymes and altered profiles of DNA methylation and demethylation.

The relationship between the redox environment and proline is complex. Proline metabolism is affected by the redox status [49] but also affects the NAD(P)^+^/NAD(P)H ratio in cytosol and mitochondria, as well as the generation of ROS [50,51], behaving, on the whole, as a redox buffer. The control of proline over ROS distribution and cellular redox status largely derives from its peculiar compartmentalized metabolism. Since proline synthesis occurs in the cytosol and proline catabolism in the mitochondrion, it is possible to generate, accumulate, and recycle reducing equivalents and ROS at different concentrations in different cellular compartments.

During proline synthesis in the cytosol, glutamate is reduced to proline by the sequential action of the P5CS and Δ^1^-pyrroline-5-carboxylate reductase (P5CR). The reactions are coupled with the oxidation of NAD(P)H to NAD(P)^+^ and are subjected to feedback inhibition by proline and other cofactors [49]. In addition to maintaining a high NAD(P)^+^/NAD(P)H ratio in the cytosol, proline synthesis feeds the pentose phosphate pathway [52], and in turn, the glutathione/ascorbate pathway, contributing to maintaining high levels of antioxidants and low levels of H_2_O_2_ [53]. During proline catabolism, proline is transported into the mitochondrion by a still-uncharacterized proline symporter or proline/glutamate antiporter and is oxidized back to glutamate by proline dehydrogenase (ProDH) and Δ^1^-pyrroline-5-carboxylate dehydrogenase (P5CDH). This process requires FADH and NAD^+^ reduction and feeds the electron transport chain to produce ATP. When the electrons generated by proline oxidation exceed the capability of the electron transport chain, however, O_2_^•−^ and H_2_O_2_ are also produced as by-products of mitochondrial respiration.

A delicate equilibrium between ATP and ROS production is maintained by multiple regulatory systems, by modulating the relative activities of ProDH and P5CDH and directing proline catabolism toward glutamate production or the proline–P5C cycle. In this cycle, the P5C generated by proline oxidation in the mitochondrion is not further oxidized to glutamate by P5CDH but re-enters in the cytosol, where it is reduced back to proline by P5CR. This seemingly futile cycle allows the transfer of reducing equivalents into the mitochondrion and the generation of ROS and may behave as a master regulator of redox balance and ROS signaling.

The classical models of gene induction involve binding either transcription factors or small regulative molecules to the promoters of target genes to regulate their transcriptional activity. Unfortunately, this model of action does not seem not to apply to proline, to the best of our knowledge. The interesting finding that proline-deficient mutants have high levels of *FLC*, however, suggests that proline may affect the epigenetic regulation of *FLC* expression by modifying the redox status of the cell.

In this communication article, we investigated the molecular basis of the late flowering in *p5cs1 p5cs2/P5CS2* mutants and showed, through molecular, physiological, and genetic experiments, that the effects on the flowering time of proline metabolism depend on FLC. Although this finding cannot explain how proline can modulate *FLC* expression, in view of the results of this study, it may suggest a possible mechanism of epigenetic regulation, which deserves further investigation. However, considering the multiple development pathways in which proline seems to be involved, we cannot rule out, at present, that proline-mediated *FLC* regulation may occur through different mechanisms acting at a transcriptional or post-transcriptional level.

## 4. Materials and Methods

### 4.1. Plant Growth Conditions, Physiological Experiments, and Plant Treatments

All the *Arabidopsis* mutants used in this work were of Columbia-0 (Col-0) ecotype and were grown in a growth chamber at 24/21 °C with a light intensity of 300 µE·m^−2^·s^−1^ under 16 h light and 8 h dark per day (under either 16 h light and 8 h dark (long days) or 8 h light and 16 h dark (short days)). Seeds were stratified for three days at 4 °C, surface-sterilized, germinated on MS1/2, and potted a week after. *p5cs1 p5cs2/P5CS2* mutants were generated by crossing a homozygous *p5cs1* mutant (Salk__063517) obtained from the Salk collection, with a heterozygous *p5cs2* mutant (GABI_452G01) obtained from the GABI-Kat collection [4]. As *p5cs2* mutants are embryo-lethal, and homozygous lines cannot be produced [3,4], only quasi-double mutants were generated. The late-flowering phenotype of the *p5cs1 p5cs2/P5CS2* quasi-double mutants was first described by Mattioli et al., (2009) [4].

To maintain the *p5cs2* mutant allele and carry out segregation analysis, heterozygous *p5cs2/P5CS2* single mutants and *p5cs1 p5cs2/P5CS2* quasi-double mutants were germinated on MS1/2 plates supplemented with 6 μg/mL sulfadiazine (sul). Vernalization experiments were performed by keeping seeds in the dark at 4 °C for three months. We performed the flowering time analysis by counting the number of rosette leaves present at the appearance of the first flower bud [2,54,55]. We carried out the trichome analysis by counting the number of trichomes under a dissection microscope. We defined juvenile leaves as rosette leaves with adaxial trichomes only, transition leaves as rosette leaves with at least one trichome located at the abaxial base of the midrib, and adult leaves as rosette leaves with abaxial trichomes distributed all over the abaxial side, both at the midrib and at the lamina of the leaf. All the experiments were performed as triplicate independent experiments, and results were expressed as average values ± SE.

### 4.2. Molecular Techniques and Real-Time RT-qPCR

Molecular techniques were performed according to standard protocols. For PCR analysis, genomic DNA was extracted with a modified CTAB method, according to Stewart and Via (1993) [56]. Real-time RT-qPCR analyses were carried out with a Rotor-Gene Q (Qiagen, Hilden, Germany). Amplifications were monitored using the SYBR Green fluorescent stain. The presence of a single PCR product was verified by a dissociation analysis in all amplifications. All the primers used for RT-PCR and RT-qPCR analyses were designed with Primer-BLAST (https://www.ncbi.nlm.nih.gov/tools/primer-blast/, accessed on 15 August 2022) and are reported in Table 1. Primer efficiency was determined for each pair of primers by amplifying serial dilutions of target genes and by plotting the resulting Cq against the log-transformed value of the dilution. The comparative threshold cycle (ΔΔCq) method was used to calculate the relative amount of gene expression and normalized using the Cq values derived for the housekeeping *ACTINE8* (*ACT*). All the analyses were performed in triplicate on, at least, three independent samples. Statistical analysis was performed on raw Ct values. Total RNA for RT-qPCR was extracted from leaves or apical shoots using a NucleoSpin RNA Plant (Macherey-Nachel, Hoerdt, France) according to the manufacturer’s instructions. To prevent mRNA decay, we dissected leaves and apices in a cold room and threw them in liquid nitrogen immediately after dissection. RNA quality (A_260_/A_280_ ratio > 2) and quantity was assessed with a NanoDrop 1000 (Thermo Fisher Scientific, Milan, Italy). All samples were taken when we saw the first floral bud in the wild-type population. Reverse transcription was performed from 1 μg of total RNA using a QuantiTect Reverse Transcription kit (Qiagen, Hilden, Germany) as recommended by the manufacturer. Plant material was collected from plants close to flower transition, with still no visible signs of flowering. To enrich tissues in SAM, apices were dissected under a Zeiss Stemi SV6 stereo-microscope.

### 4.3. Identification and Characterization of Flc-7 T-DNA Insertion Mutant

A T-DNA insertion mutant in a Col-0 background was identified in the GABI-KAT collection [44] and obtained from the NASC stock center (GK 145D01). Seed samples of T2 progeny were germinated under sulfadiazine selection and screened by PCR for the presence of T-DNA insertion using FLC- and T-DNA-specific primers (Table S1). PCR conditions were as follows: 3′ at 94 °C followed by 33 cycles of 45″ at 94 °C, 45″ at 60 °C and, 1′30″ at 72 °C. For RT-PCR, the total RNA was extracted from leaves using Trizol reagent (Invitrogen) according to the manufacturer’s instructions. Reverse transcription was performed from 1 μg of total RNA using a QuantiTect Reverse Transcription kit (Qiagen, Hilden, Germany) as recommended by the manufacturer.

### 4.4. Plant Genetic Crosses

Since *p5cs1 p5cs2/P5CS2* mutants cannot transmit pollen grains bearing the *p5cs2* mutant allele [6], genetic crosses between *p5cs1 p5cs2/P5CS2* and *flc-7* were made using the former as female and the latter as male. Seeds from outcrossed siliques were grown under sulfadiazine selection, and resistant plantlets were checked by using PCR to identify individuals heterozygous for all the mutations. Seeds from self-fertilized F2 plants were grown under sulfadiazine selection, and resistant plantlets were checked by using PCR to identify individuals heterozygous for *p5cs2* and homozygous for *p5cs1* and *flc-7*.

## Figures and Tables

**Table 1:** List of the flowering time genes analyzed.

Full Gene Name	Acronym	AGI Locus Code	Floral Pathway
*GIGANTEA*	*GI*	AT1G22770	Photoperiodic
*FLOWERING WAGENINGEN*	*FWA*	AT4G25530	Photoperiodic
*CONSTANS*	*CO*	AT5G15840	Photoperiodic
*LUMINIDEPENDENS*	*LD*	AT4G02560	Autonomous
*FLOWERING CONTROL LOCUS A*	*FCA*	AT4G16280	Autonomous
*FLOWERING LOCUS KH DOMAIN*	*FLK*	AT3G04610	Autonomous
*FLOWERING LOCUS D*	*FLD*	AT3G10390	Autonomous
Unknown	*FPA*	AT2G43410	Autonomous
Unknown	*FVE*	AT2G19520	Autonomous
Unknown	*FY*	AT5G13480	Autonomous
*REDUCED VERNALIZATION RESPONSE 1*	*VRN1*	AT3G18990	Vernalization
*REDUCED VERNALIZATION RESPONSE 2*	*VRN2*	AT4G16845	Vernalization
*VERNALIZATION INSENSITIVE 3*	*VIN3*	AT5G57380	Vernalization
*GA REQUIRING 1*	*GA1*	AT4G02780	GA-dependent
*GIBBERELLIN 3-OXIDASE 1*	*GA3ox1*	AT1G15550	GA-dependent
*GIBBERELLIN 3-OXIDASE 2*	*GA3ox2*	AT1G80340	GA-dependent
*GIBBERELLIN 3-OXIDASE 3*	*GA3ox3*	AT4G21690	GA-dependent
*GIBBERELLIN 3-OXIDASE 4*	*GA3ox4*	AT1G80330	GA-dependent
*GIBBERELLIN 20 OXIDASE 1*	*GA20ox1*	AT4G25420	GA-dependent
*GIBBERELLIN 20 OXIDASE 2*	*GA20ox2*	AT5G51810	GA-dependent
*GIBBERELLIN 2-OXIDASE 1*	*GA2ox1*	AT1G78440	GA-dependent
*FLOWERING LOCUS T*	*FT*	AT1G65480	Floral integrator
*FLOWERING LOCUS C*	*FLC*	AT5G10140	Floral integrator
*SUPPRESSOR OF OVEREXPRESSION OF CO1*	*SOC1*	AT2G45660	Floral integrator
*LEAFY*	*LFY*	AT5G61850	Floral integrator
*APETALA 1*	*AP1*	AT1G26310	Meristem identity
*CAULIFLOWER*	*CAL*	AT1G26310	Meristem identity

Note. The *FVE* gene is also known as *MSI4*. The *FY* gene is also known as *WDR33*. *LFY* also behaves as a floral meristem identity gene.

## Data Availability

Not applicable.

## Supplementary Materials

The following supporting information can be downloaded at: https://www.mdpi.com/article/10.3390/plants11182348/s1, Table S1: List of PCR primers used in this work.
