# Supplementary material for: Proline Affects Flowering Time in Arabidopsis by Modulating FLC Expression: A Clue of Epigenetic Regulation?
Source: Plants (Basel). 2022 Sep 8;11(18):2348. doi: 10.3390/plants11182348 (PMC9505445; doi:10.3390/plants11182348)
Supplement: Supplementary file 1 [file plants-11-02348-s001.zip › plants-1894612-supplementary.pdf]

Table S1: List of PCR primers used in this work:

Flowering time			
CO for:	tcgtggctgttccctaattc	*CO* rev:	ccctgaggagccatatttga
GI for:	gagctgtctttctccgttgttt	*GI* rev:	cttcaatagattggataaaccgtc
FWA for:	tatgcaccagtggaaaccaa	*FWA* rev:	gagtgcagcagttggattga
LD for:	atggaccgcgttcaaggaggagata	*LD* rev:	acatgcctccggatgtatagagtt
FPA for:	atgggaaaggggattgaaac	*FPA* rev:	ctgatggaggcaccaagaat
FVE for:	catcacaacgattgggacag	*FVE* rev:	aaccttcgtgacagggtttg
FCA for:	catacctcgcctgatggatt	*FCA* rev:	aaacggctgctgtaattgct
FY for:	caagttttggtgcagaaacagg	*FY* rev:	tgttgttgctgatacccttgct
FLK for:	aggggagtacctggtgaaatga	*FLK* rev:	ggcatctagcgtttccactctt
FLD for:	taggaacccatcaaggaatgct	*FLD* rev:	actgaatcagctccaacaccaa
LFY for:	acgaaggtgaggatgacgac	*LFY* rev:	catttttcgccacggtctttag
SOC1 for:	aattcgccagctccaatatg	*SOC1* rev:	cctcgattgagcatgttcct
FT for:	ctggaacaacctttggcaat	*FT* rev:	agccactctccctctgacaa
FLC for:	cggtctcatcgagaaagctc	*FLC* rev:	tagtcacggagagggcagtc
VRN1 for:	gggtatgctagttcagccatcc	*VRN1* rev:	acttctcagcaaacccagaagg
VRN2 for:	gcttgctggacaatttgagatg	*VRN2* rev:	aacgagttccaaagatgcatga
VIN3 for:	agcaaaccctttgacaaactc	*VIN3* rev:	tttgcggcattattgatctcag
GA1 for:	gtctcgcggaaatcatcaat	*GA1* rev:	tgatcgccacataaagcaaa
GA3ox1 for:	cgatttccgtaaactttggc	*GA3ox1* rev:	atccattggataggatgtgg
GA3ox2 for:	ggcgtagctcgtattgcttc	*GA3ox2* rev:	tggataactgcttgggttcc
GA3ox3 for:	ttcgacgtcacgctacactc	*GA3ox3* rev:	caaaccgggtaggagttcaa
GA3ox4 for:	cctgacgttaccaccctcat	*GA3ox4* rev:	ttcaatgtcttccacggtga
GA20ox1 for:	aaatccggtgagagtgttgg	*GA20ox1* rev:	cttagccccagaagctccat
GA20ox2 for:	tgccaaacaccagatctcac	*GA20ox2* rev:	tcgcgctctctctattcaca
GA2ox1 for:	atcaatggcggtattgtctaaac	*GA2ox1* rev:	accttgaagaagccgaagtc
AP1 for:	gcaagcaatgagccctaaag	*AP1* rev:	actgctcctgttgagcccta
CAL for:	gaaccaatgagcctcaagga	*CAL* rev:	ttgtggctgtggtacatcgt
ACT for:	atgaagattaaggtcgtggca	*ACT* rev:	Tccgagtttgaagaggctac
Flowering time (RT-qPCR)			
CO for:	cacgaccctgtgacacatgc	*CO* rev:	catggcaatacacggtgcag
SOC1 for:	aacgtaaactcttgggagaaggc	*SOC1* rev:	gcagctcctcgattgagcat
FT for:	cttggcaggcaaacagtgtatg	*FT* rev:	agtgttgaagttctggcgcc
FLC for:	atgtgggagcagaagctgaga	*FLC* rev:	cggagatttgtccagcaggt
GA1 for:	ttcatgcagacccgagacagt	*GA1* rev:	gacggcatttcgcaaatactc
LFY for:	tcctgaaggtttcacgagtgg	*LFY* rev:	aatgctctcgttgggttcca
AP1 for:	ccacaatatgcctccccctc	*AP1* rev:	ggatgctggatttggtgctg
CAL for:	tgatgccgaggtttccctta	*CAL* rev:	tcgaacaatttgcccttatgg
ACT for:	cttgcaccaagcagcatgaa	*ACT* rev:	ccgatccagacactgtacttcctt

Characterization of *flc-7* and *p5cs1 p5cs2*/*P5CS2*			
FLC for:	caaacaaagagctgatgttaccaa	*FLC* rev:	tagtcacggagagggcagtc
FLC for:	caaacaaagagctgatgttaccaa	*T-DNA* rev:	ataataacgctgcggacatctacatttt
P5CS1 for:	ggaacccaccatttgcatttgtgt	*P5CS1* rev:	tctgcaacttcgtgatcctctgtc
P5CS1 for:	ctgttgggggtaaactcattg	*LBb1* rev:	gcgtggaccgcttgctgcaact
P5CS2 for:	ggagcagaatggttttctcg	*P5CS2* rev:	tggaaaacagcagcactgtc
P5CS2 for:	ggagcagaatggttttctcg	*T-DNA* rev:	tatctgggaatggcgaaatc
